# Experimental Investigation on Process Parameters during Laser-Assisted Turning of SiC Ceramics Based on Orthogonal Method and Response Surface Methodology

**DOI:** 10.3390/ma15144889

**Published:** 2022-07-14

**Authors:** Di Dai, Yugang Zhao, Chen Cao, Ruichun Dong, Haiyun Zhang, Qian Liu, Zhuang Song, Xiajunyu Zhang, Zhilong Zheng, Chuang Zhao

**Affiliations:** School of Mechanical Engineering, Shandong University of Technology, Zibo 255049, China; dd1226sdut@126.com (D.D.); caochen@sdut.edu.cn (C.C.); dongrcn@163.com (R.D.); liuqian@sdut.edu.cn (Q.L.); sdut603315@163.com (Z.S.); sjxpf718ner77@163.com (X.Z.); zzlsdut@126.com (Z.Z.); zhaochuangsdut@126.com (C.Z.)

**Keywords:** laser-assisted turning, SiC ceramics, orthogonal method, response surface methodology

## Abstract

In this study, laser-assisted machining experiments are carried out on silicon carbide (SiC) ceramic materials by a turning process, and laser power, cutting depth, rotational speed, and feed speed are selected as research factors. In order to improve the surface processing quality of laser-assisted turning of SiC ceramics and obtain the smallest surface roughness, the orthogonal method and response surface method are used to investigate the effect of various factors on surface roughness. The effect of various factors on surface roughness is evaluated by variance analysis, mean analysis, main effect diagram, 3D response surface, and corresponding contour diagram. The surface roughness prediction model is established based on the response surface method, and the prediction error is 4.1% with high accuracy. The experimental results show that laser power and cutting depth are the most significant factors affecting surface roughness, and rotational speed is a significant factor affecting surface roughness. Under the optimum process conditions, the smallest surface roughness *Ra* obtained by the response surface method is 0.294 μm, which is lower than 0.315 μm obtained by the orthogonal method, and the surface quality is higher. Therefore, the optimal process parameters of the response surface method can obtain the smallest surface roughness and higher surface quality in laser-assisted turning of SiC ceramics.

## 1. Introduction

Silicon carbide (SiC), as one of the engineering ceramics, and because of its high wear resistance, high corrosion resistance, and high temperature strength, is used for various wear-resistant, corrosion-resistant, and high-temperature resistant mechanical parts. However, due to the high hardness and brittleness of SiC ceramics, serious defects easily occur in the processing, resulting in poor surface quality, which affects the serviceability of the parts [1,2]. In the absence of any auxiliary technology, it is difficult to obtain high-quality surfaces of SiC ceramics, which is also one of the important reasons for the large-scale application of SiC ceramics.

With the rapid development of modern manufacturing and processing technology, the emergence of laser-assisted machining technology has overcome the processing problems of difficult-to-machine materials and has received extensive attention from the outside world. Laser-assisted machining technology combines traditional cutting and laser heating, where the laser is used to heat the material in the cutting area directly in front of the tool to reduce the strength of the material in this area and to form a softening layer, and the material is then removed by conventional cutting [3,4]. Compared with the traditional processing methods of turning, grinding, and milling, the processing efficiency and processing quality of laser-assisted machining technology have been significantly improved, and are also more in line with the concept of energy saving, which has become the focus of current world research [5,6,7,8]. König et al. applied the laser-assisted machining process to the processing of Si_3_N_4_ ceramic materials for the first time, and the results showed that when the temperature reaches 1200 °C, continuous chips similar to metal cuttings were obtained during cutting [9]. Lei et al. investigated the material deformation behavior of silicon nitride ceramics containing a 10% YSiAlON glass phase under a laser-assisted machining process and researched the aspects of tool wear, material removal mechanisms, and machined surface integrity, which showed that laser-assisted machining is an economical and feasible process for manufacturing precision ceramic parts [10]. Bejjani et al. studied the laser-assisted turning of titanium metal, and through the analysis of chip state and microstructure, it was found that laser-assisted turning technology can increase the tool life by 180% [11]. Zahrani et al. carried out laser-assisted turning of carbon steel and analyzed the chips formed under different cutting conditions and found that laser-assisted turning not only can actually shorten the chip, but also greatly reduce the cutting force and surface roughness [12]. Przestacki carried out laser-assisted turning for metal matrix composites and studied the influence of the laser beam on cutting force, tool wear, and surface roughness during turning, and the results showed that the quality of laser-assisted machining is much higher than that of traditional turning [13]. Dhupal et al. investigated the relationship between the process parameters of laser-assisted turning of alumina ceramics, and the analysis found that proper control of process parameters could obtain good processing results [14]. Omid carried out a laser-assisted machining experiment and a conventional machining experiment on a Ti-6Al-4V alloy, respectively, and compared and discussed the experimental results of surface integrity which showed that laser-assisted machining could greatly improve the surface machining quality of the Ti-6Al-4V alloy [15]. Chang evaluated the economic feasibility of laser-assisted turning as the manufacturing process of precision alumina ceramic parts, and analyzed the surface roughness by the Taguchi method, which showed that laser-assisted machining could obtain a better workpiece surface quality, a greater material removal rate, and moderate tool wear than traditional machining [16]. Based on the Taguchi method, Venkatesan et al. carried out experimental research on laser-assisted turning of Inconel 718, and analyzed the effect of cutting speed, feed speed, and laser power on cutting force, and found that under the optimal cutting conditions, the cutting force of laser-assisted turning decreased by 60% at most [17]. Song et al. investigated the effect of spindle speed, feed speed, cutting depth, and laser pulse duty ratio on the cutting force of laser-assisted turning fused quartz based on the Taguchi method and RSM, and the experimental results showed that the optimal parameter combination of RSM generates a smaller cutting force and better surface integrity in the process of laser-assisted turning of fused silica [18]. Zhai et al. conducted laser-assisted micromachining experiments on carbon fiber reinforced silicon carbide matrix (C/SiC) composites based on the response surface method, and investigated the effect of laser power, cutting speed, and cutting depth on cutting force, and obtained the mathematical prediction model of cutting force F_x_, F_y_, F_z_ in three directions in the cutting process [19]. Conventional turning and laser-assisted turning experiments on SiC-Al2O3 reinforced aluminum hybrid nanocomposites were carried out by Reza et al., and the laser power, cutting depth, and cutting speed were optimized based on the Taguchi method to find the smallest surface roughness and cutting force. The results showed that the cutting speed is the most significant factor affecting the surface roughness during laser-assisted turning, and under the optimal processing conditions, compared with conventional turning, the surface roughness and cutting force obtained by laser-assisted turning are reduced by 51% and 26%, respectively [20].

Combined with previous studies, laser-assisted machining technology as the main processing method of difficult-to-machine materials can effectively reduce the cutting force and surface roughness in the process of machining. However, there is little research on ceramic material turning under the condition of a high-power continuous laser, because when other process parameters are not properly selected, the high-power continuous laser can easily cause thermal damage to the workpiece, resulting in poor machining quality. Therefore, in this study, the high-power continuous laser-assisted machining experiments of SiC ceramic materials are carried out by the turning process, and the effect of laser power, cutting depth, rotational speed, and feed speed on surface quality is discussed. With the smallest surface roughness as the objective, the orthogonal method and RSM are used to optimize the objective, respectively, and the optimal combination of process parameters and the optimal surface quality are obtained by comparing the experimental results. The orthogonal method will select representative points from the comprehensive experiment according to the orthogonality, which can effectively find out the optimal process parameters of each factor. RSM realizes accurate prediction of surface roughness by establishing a response surface regression model, and the interaction between process parameters and surface roughness is analyzed, and the optimal combination of process parameters is obtained. This study has important guiding significance for laser-assisted turning of SiC ceramics.

## 2. Experiments

### 2.1. Experimental Equipment and Materials

**Figure 1:**
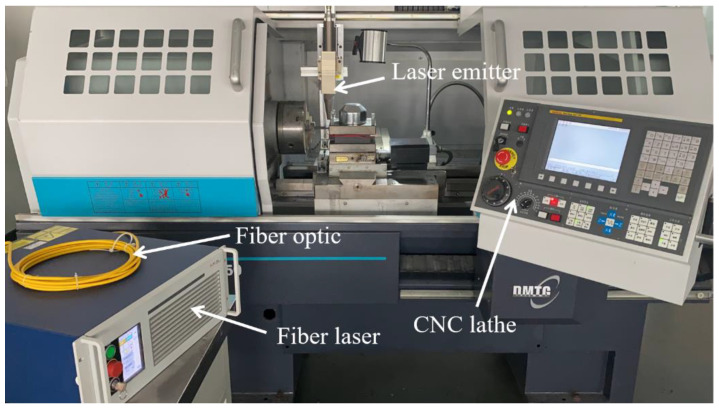
Laser-assisted turning equipment.

Figure 1 shows the laser-assisted turning equipment, which mainly includes a CNC lathe and laser-assisted heat source system. A CNC lathe (CKD6136i, Dalian Machine Tool Group, Dalian, China) with a FANUC control system, the maximum workpiece turning diameter of 360 mm, and the maximum clamping length of 1000 mm was used; the laser-assisted heat source system includes a fiber laser and laser emitter. The fiber laser (YLR-150/1500-QCW-MM-AC-Y14, IPG Photonics Corporation, Oxford, MS, USA) has two modes of pulse and continuous, and the maximum average power of the continuous laser was 250 W, and the typical wavelength was 1070 nm. The surface roughness of the machined area was measured several times using a 3D digital microscope (DSX1000, OLYMPUS, Tokyo, Japan), and the final surface roughness results were averaged from the multiple measurements. The experimental material was a SiC ceramic bar with a specification of Φ11 × 150 mm, and the laser absorption rate of the material at a typical laser wavelength was about 0.75 [21], and its main mechanical and thermal properties are shown in Table 1.

### 2.2. Experimental Principle

**Figure 2:**
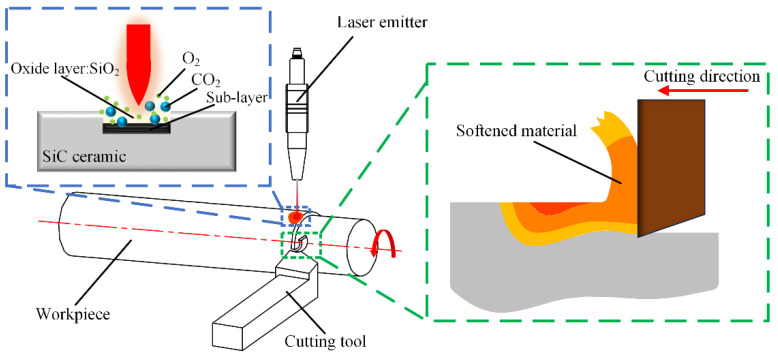
Principle of laser-assisted turning.

Figure 2 shows the principle of laser-assisted turning. Laser-assisted turning technology combines conventional turning with laser heating. During processing, the continuous laser is irradiated on the surface of the workpiece, the laser is heated before, and the tool is cut after. Before the material is removed, the high energy of the laser makes the temperature of the area to be processed rapidly rise, the material strength decreases, and the plasticity increases. Finally, the material is removed by the tool [22,23]. Compared with conventional turning, laser-assisted turning can effectively reduce the cutting force, reduce the friction between the tool and the machined surface, reduce the surface roughness, and effectively improve the surface machining quality [24,25].

### 2.3. Experimental Design

#### 2.3.1. Orthogonal Experiment

In the experiment of laser-assisted turning of SiC ceramics, the surface processing quality is usually affected by many factors. Since the laser power, cutting depth, rotational speed, and feed speed are the main process parameters, the above process parameters were selected as the orthogonal experimental factors.

The orthogonal experimental factor levels were determined by the results of a series of previous single-factor experiments, and the specific experimental levels: laser power 210~240 W, cutting depth 0.10~0.20 mm, rotational speed 1500~1740 r/min, feed speed 2~4 mm/min. The L9 (3^4^) orthogonal experimental scheme with 4 factors and 3 levels was designed with the surface roughness *Ra* value as the evaluation index, and the specific experimental scheme is shown in Table 2.

#### 2.3.2. Response Surface Experiment

RSM is a statistical analysis method based on mathematical and experimental data, which is used to solve the optimization problem of multiple variables [26]. By constructing the response surface model between each experimental factor and the response value, the functional relationship between the response target and the design variables is established to achieve the optimal solution for the response value.

As an experimental method based on response surface theory, the Box-Behnken design (BBD) experiment is widely used in various experiments [27]. On the basis of single factor experiment analysis, laser power, cutting depth, rotational speed, and feed speed were selected as optimization parameters, and were set as independent variables *A*, *B*, *C*, and *D* in turn, and each independent variable was selected at −1, 0, and + 1 levels. Taking the surface roughness *Ra* value of the machined SiC workpiece as the corresponding response index, a four-factor and three-level BBD experimental scheme was designed, as shown in Table 3.

## 3. Results and Discussion

### 3.1. Orthogonal Experimental Results and Analysis

Table 4 shows the results of the orthogonal experiment, indicating that the surface roughness *Ra* value of the processed SiC is in the range of 0.321~0.765 μm. In order to investigate the degree of effect of laser power, cutting depth, rotational speed, and feed speed on the surface roughness and to obtain the optimal combination of process parameters, range analysis and variance analysis were carried out for the experimental results in Table 4 [28,29].

#### 3.1.1. Analysis of Variance of Surface Roughness

Based on the experimental results of surface roughness in Table 4, variance analysis was used to investigate the influence of various factors on surface roughness. Since there is no blank column in the designed orthogonal table, the least square sum of the four factors was used as the source of the error square sum, and the feed speed was selected as the error source [30].

Table 5 shows the variance analysis results of surface roughness, in which the F- value of cutting depth is 298.85, which indicates that the variation of the cutting depth has an extremely significant effect on the surface roughness within the determined parameters. The F-value of laser power is 84.30, which indicates that the variation of the laser power has a significant effect on the surface roughness within the determined parameters. The F-value of the rotational speed is 16.68, which indicates that the variation of the rotational speed has a slightly significant effect on the surface roughness within the determined parameters. The variation of feed speed has a low effect on surface roughness. The main reason for the extremely significant effect of cutting depth on surface roughness is that SiC ceramics have the characteristics of high hardness and high brittleness, the laser is the only heat source for softening SiC materials, and its power determines the depth of the softening layer of the material. When the cutting depth is greater than the softening layer depth, the surface roughness will rise sharply.

#### 3.1.2. Range Analysis of Surface Roughness

Table 6 shows the results of surface roughness range analysis, and the results show that the range *R* value of laser power, cutting depth, rotational speed, and feed speed are 0.1647, 0.3033, 0.0733, and 0.0177, respectively. By comparing the corresponding *R* value of each factor, it was found that the corresponding *R* value of cutting depth was the largest, indicating that the effect of cutting depth on surface roughness is extremely obvious. The corresponding *R* value of feed speed was the smallest, indicating that the effect of feed speed on surface roughness is not obvious. The effect of four factors on surface roughness is ranked as follows: cutting depth (a_p_) > laser power (P) > rotational speed (n) > feed speed (f).

**Figure 3:**
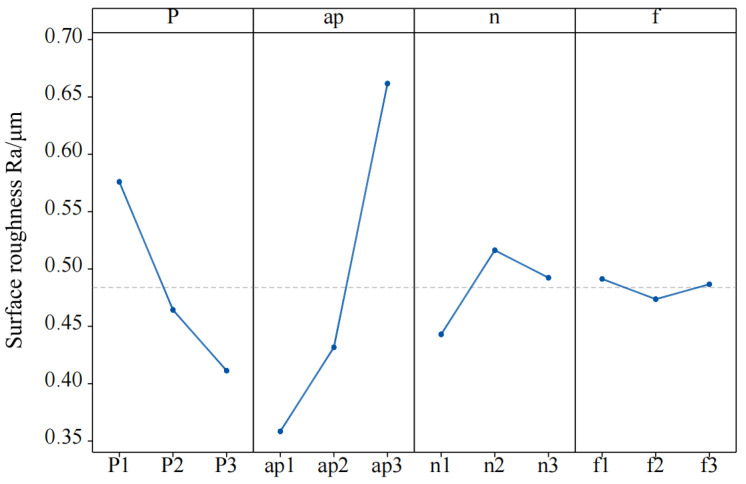
Main effect change of mean surface roughness.

Figure 3 shows the plot of the mean effect of each factor level with surface roughness obtained based on the results of the range analysis, and the variation trend of surface roughness can be directly reflected in the level range of each factor. The optimization level combination was determined by analyzing the variation trend of surface roughness in Figure 3. The specific optimization parameters were: laser power 240 W, cutting depth 0.1 mm, rotational speed 1500 r/min, feed speed 3 mm/min. Using this optimized combination of parameters for cutting SiC ceramics, the surface roughness *Ra* value of 0.315 μm was obtained, which is lower than that of 0.321 μm in experiment No. 8 of the orthogonal scheme.

### 3.2. Response Surface Regression Model

The surface roughness *Ra* of the processed SiC workpiece was used as the response index, and the results of the BBD experiment are shown in Table 7. Based on the experimental results, the regression model equations of laser power, cutting depth, rotational speed, and feed speed with respect to surface roughness are established by Design Expert (Equation (1)).

(1) $$\begin{array}{r} Ra=0.4033+0.0899A+0.1531B+0.0168C-0.0029D-0.0223AB-0.0108AC-0.0112AD\\ +0.0207BC-0.0032BD+0.0008CD+0.0178A^{2}+0.0948B^{2}+0.0133C^{2}+0.0128D^{2} \end{array}$$

**Figure 4:**
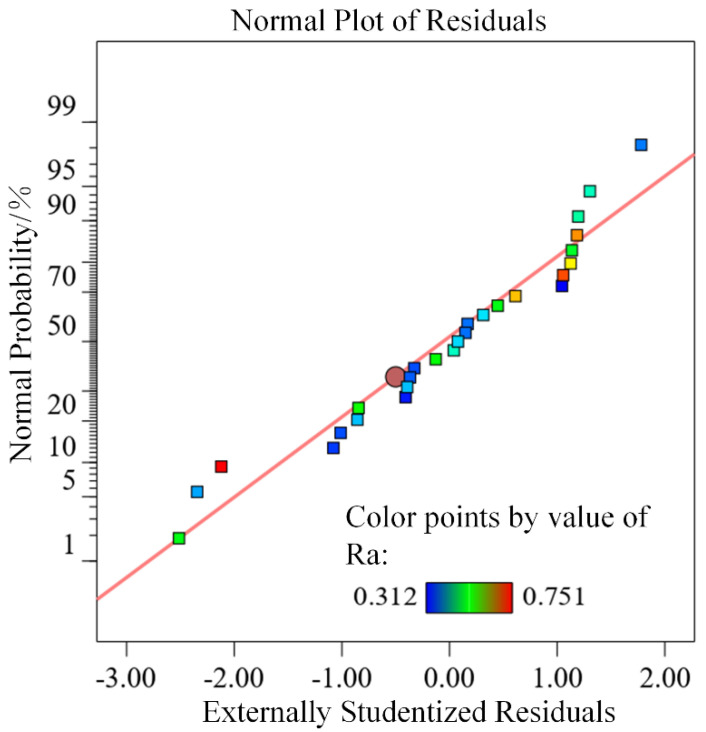
Residual normal distribution.

Figure 4 shows the normal distribution of the surface roughness prediction model residual. By observing Figure 4, it is found that the residuals are basically distributed on a straight line, which indicates that the predicted and actual values of the model of surface roughness are in high agreement, and the accuracy of the predicted model of each process parameter and surface roughness is high.

#### 3.2.1. Analysis of Variance of Regression Model

Table 8 shows the results of variance analysis of surface roughness, and the results show that the F-value of the model is 49.85 and the *p*-value is inferior to 0.0001, which indicates that the regression model established between each variable and surface roughness is highly significant. The F-value of the lack of fit item is 12.71, the *p*-value is higher than 0.05, the lack of fit item is not significant, and the model coefficient of determination *R*^2^ = 0.9831, which indicates that the regression model fits well in the whole regression area, with high reliability and accuracy, and small error. Among the four factors affecting the surface roughness, the *p*-value of laser power (*A*) and cutting depth (*B*) is inferior to 0.0001, which indicates that laser power (*A*) and cutting depth (*B*) have highly significant effects on the surface roughness. The *p*-value of rotational speed (*C*) is inferior to 0.05, which indicates that the rotational speed (*C*) has a significant effect on the surface roughness. The *p*-value of feed speed (*D*) is higher than 0.05, which indicates that the effect of feed speed (*D*) on surface roughness is not significant. By comparing the mean square values of the respective variables, it can be seen that the effect on the surface roughness is in the order of: *B* > *A* > *C* > *D.* Among the secondary terms, the highly significant effect is *B*^2^. Among the interaction terms, the interaction effect of *AB*, *BC*, and *AD* is significant.

#### 3.2.2. Interaction Analysis of Surface Roughness

**Figure 5:**
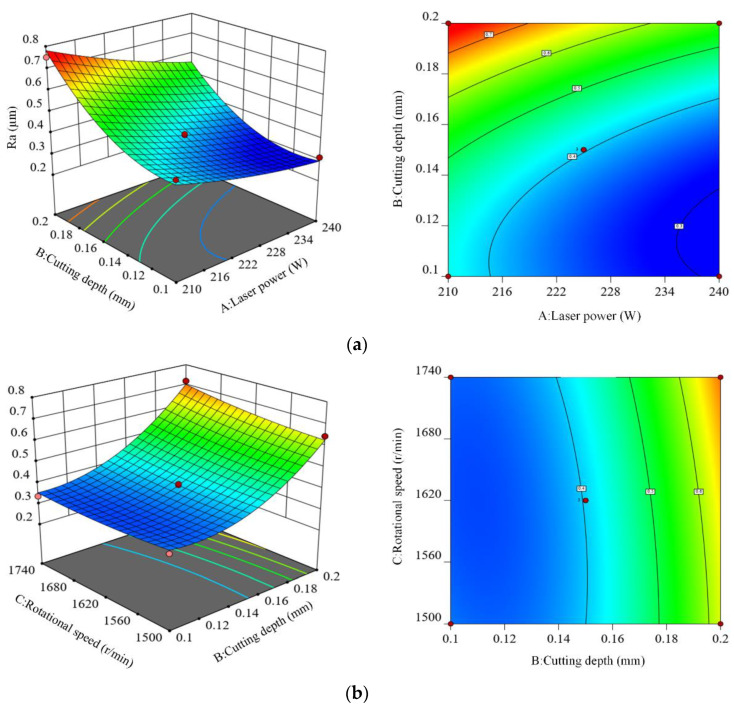

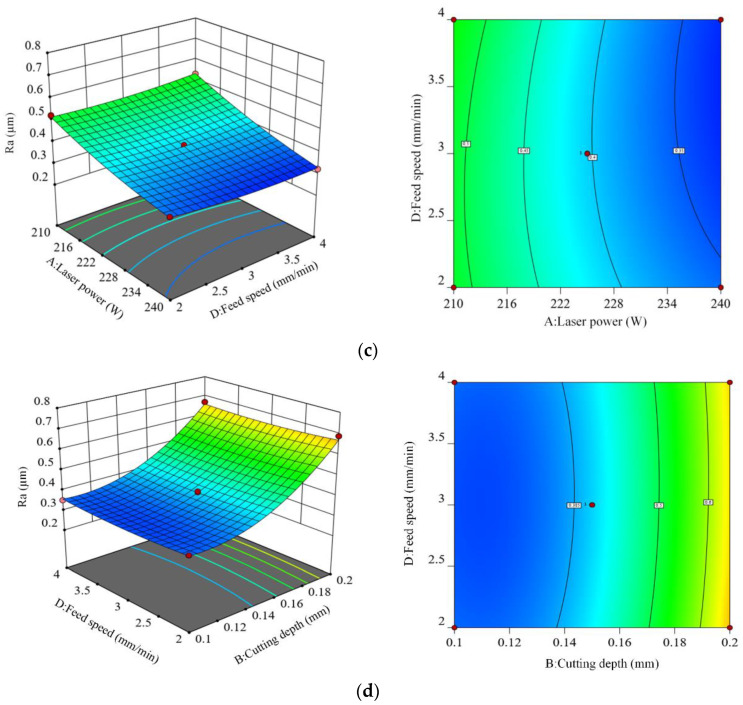
Three-dimensional surface and contour diagram. (**a**) Interaction of laser power and cutting depth, (**b**) Interaction of cutting depth and rotational speed, (**c**) Interaction of laser power and feed speed, (**d**) Interaction of cutting depth and feed speed.

Figure 5 describes the interaction effect of various process parameters on surface roughness and generates 3D surfaces and corresponding contour diagrams based on the regression model. The 3D curve profile makes it easier to understand the interaction between different combinations of factors; the change in curvature of the contour can determine whether the interaction between two independent factors is significant.

Figure 5a shows the 3D surface figure and the corresponding contour diagram of the interaction between laser power (*A*) and cutting depth (*B*) on surface roughness. The variation trend of surface roughness in the figure shows that when the rotational speed and feed speed are fixed at the intermediate level, the surface roughness tends to increase with the increase of cutting depth. In addition, the surface roughness shows a decreasing trend with the increase of laser power. Therefore, the way to obtain the smallest surface roughness is to use higher laser power and smaller cutting depth. The reason is that the laser is the only heat source, and its power determines the softening degree of the material in the turning area. When the laser power increases, the softening layer in the cutting area increases, which leads to the increase of the cutting depth, and so the surface roughness shows a decreasing trend. A better cutting depth of 0.1~0.15 mm is obtained by analyzing the contour distribution in Figure 5a, and the selection of laser power depends on the cutting depth; the laser power is 210~220 W for the smaller cutting depth, and 220~240 W for the larger cutting depth.

Figure 5b shows the 3D surface figure and the corresponding contour diagram of the interaction between cutting depth (*B*) and rotational speed (*C*) on surface roughness. The variation trend of surface roughness in the figure shows that when the laser power and feed speed are fixed at the intermediate level, the surface roughness increases with the increase of cutting depth and rotational speed. Therefore, the way to obtain the smallest surface roughness is to adopt a smaller cutting depth and rotational speed. The reason is that when the laser power and feed speed are fixed at the intermediate level, the rotational speed determines the relative motion speed of the heat source and the workpiece, and the increase of rotational speed will shorten the laser irradiation time of the turning area, and the material softening is insufficient, which leads to the decrease of the cutting depth. If the cutting depth increases, the surface roughness increases sharply. By analyzing the contour distribution in Figure 5b, the smallest surface roughness can be obtained when the cutting depth is 0.1~0.15 mm and the rotational speed is 1500~1620 r/min.

Figure 5c shows the 3D surface figure and the corresponding contour diagram of the interaction between laser power (*A*) and feed speed (*D*) on surface roughness. Under the condition that the cutting depth and the rotational speed are fixed at an intermediate level, when the laser power is at the lowest or highest level, the variation of feed speed almost does not cause the variation of surface roughness, indicating that the effect of feed speed on surface roughness is not significant. When the feed speed is at the lowest or highest level, the increase of laser power always leads to the increase of surface roughness. Therefore, the feed speed cannot be used as the basis for the selection of laser power.

Figure 5d shows the 3D surface figure and the corresponding contour diagram of the interaction between cutting depth (*B*) and feed speed (*D*) on surface roughness. The variation trend of surface roughness in the figure shows that when the laser power and rotational speed are fixed at the intermediate level, the surface roughness increases with the increase of cutting depth. In addition, with the increase of feed speed, the surface roughness decreases first and then increases. The reason is that when the feed speed is too fast, the heat source moves fast, the heating time of the turning area is short, and the material softening is insufficient. When the feed speed is too slow, the turning area is continuously irradiated by the laser, and the probability of thermal damage on the workpiece surface is large. By analyzing the contour distribution in Figure 5d, the smallest surface roughness can be obtained when cutting depth is 0.1~0.15 mm and feed speed is 2.5~3.5 mm/min.

### 3.3. Optimization and Validation

#### 3.3.1. Process Parameter Optimization

In order to obtain the optimal process parameters of laser-assisted turning of SiC ceramics, the smallest surface roughness is obtained by response surface optimization methodology. The results show that the optimal combination of process parameters are laser power 240 W, cutting depth 0.11 mm, rotational speed 1659 r/min, and feed speed 3.5 mm/min. At this point, the predicted average surface roughness *Ra* value is 0.282 μm.

#### 3.3.2. Regression Model Validation

In order to verify the accuracy of the established regression model, the obtained optimal combination of process parameters was used for three verification experiments. The final results of surface roughness take the average of experimental results. The comparison results between the predicted values of the regression model and the experimental values are shown in Table 9. The results show that the error between the experimental surface roughness value and the predicted value of the regression model is 4.1%, and Equation (1) can successfully predict surface roughness.

### 3.4. Discussion on Surface Morphology

**Figure 6:**
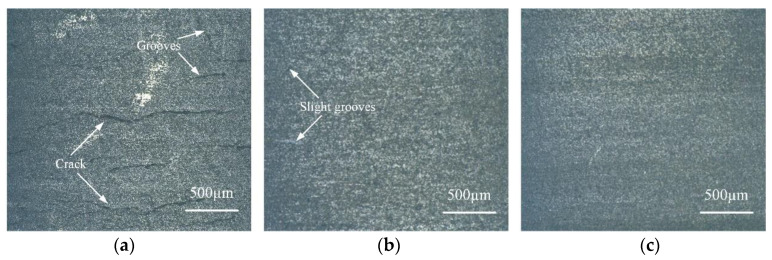
Comparison of surface morphology of SiC. (**a**) Traditional turning surface, (**b**) Surface optimization by orthogonal method, (**c**) Surface optimization by RSM.

Figure 6 shows the surface profile of the SiC workpiece obtained by turning under different process conditions. Figure 6a is the surface morphology of the SiC workpiece obtained by traditional turning with the process parameters of cutting depth 0.11 mm, rotational speed 1659 r/min, and feed speed 3.5 mm/min, and it can be observed that the surface damage traces are obvious, serious cracks are distributed, and the surface quality is poor, and the surface roughness is above 1 μm. Figure 6b shows the surface profile of the workpiece obtained by turning under laser-assisted conditions with the combination of orthogonal optimized process parameters of laser power 240 W, cutting depth 0.1 mm, rotational speed 1500 r/min, and feed speed 3 mm/min. Compared with Figure 6a, there is no obvious damage trace on the surface, and serious cracks disappear, but there are pits and bumps on the surface, the surface flatness is general, and the surface roughness is generally around 0.32 μm. Figure 6c shows the surface profile of the workpiece under laser-assisted conditions with the combination of response surface optimized process parameters of laser power 240 W, cutting depth 0.11 mm, rotational speed 1659 r/min, and feed speed 3.5 mm/min. Compared with Figure 6a and 6b, the surface damage traces disappear, the cracks disappear, the pits and bumps basically disappear, the surface flatness is high, the surface roughness is reduced to about 0.29 μm, and the surface quality is significantly improved.

**Figure 7:**
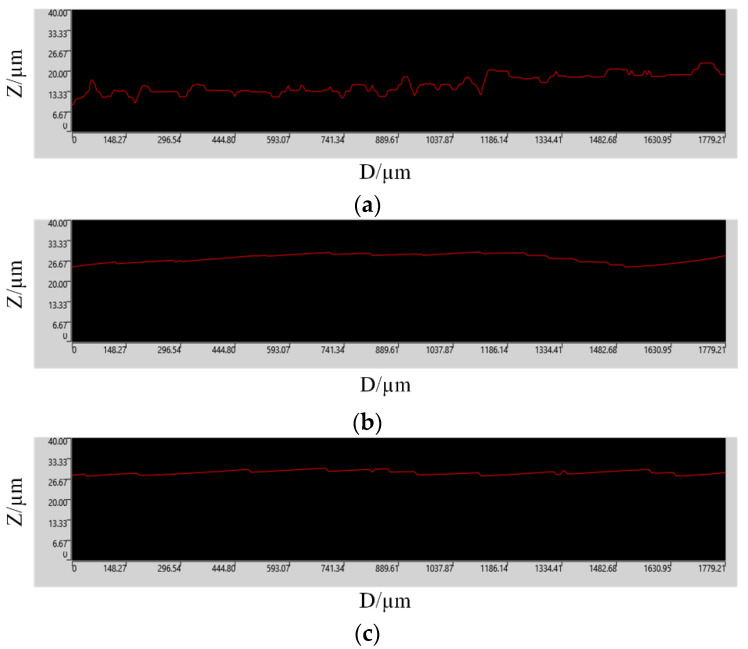
Surface roughness of SiC workpiece. (**a**) Non-auxiliary turning experiment, (**b**) Orthogonal optimization experiment, (**c**) Response surface optimization experiment.

Figure 7 shows the surface roughness measured by a DSX1000 3D digital microscope for the surface morphology of the SiC workpiece in Figure 6. By comparing the measurement results in Figure 7, the conclusions are as follows: The surface roughness obtained by a traditional turning process without any auxiliary conditions is much larger than that by a laser-assisted turning process. The appropriate combination of process parameters in the laser-assisted turning process can obtain the smallest surface roughness and significantly improve the surface processing quality.

## 4. Conclusions

In this study, the effect of process parameters on the surface quality of laser-assisted turning of SiC ceramics was studied by the orthogonal method and RSM, and a prediction model of laser-assisted process parameters on surface roughness was established. The specific conclusions are summarized as follows:

(1) According to the variance, range, and mean analysis, laser power and cutting depth are the dominant factors affecting surface roughness. The optimum parameters of the smallest surface roughness determined by the orthogonal method are laser power P 240 W, cutting depth a_p_ 0.1 mm, rotational speed n 1500 r/min, and feed speed f 3 mm/min. The actual surface roughness *Ra* value is 0.315 μm under this parameter combination.
(2) The regression model of surface roughness is established based on the RSM, and the results of variance analysis show that the model can explain 96% of the response value, with high reliability and accuracy, and statistical significance. The 3D surface and corresponding contour maps show that the interactions between laser power and cutting depth, laser power and feed speed, and cutting depth and rotational speed have a significant effect on surface roughness.
(3) Based on the RSM, the optimal process parameters are obtained as follows: laser power P 240 W, cutting depth a_p_ 0.11 mm, rotational speed n 1659 r/min, and feed speed f 3.5 mm/min. At this time, the predicted *Ra* value is 0.282 μm and the actual *Ra* value is 0.294 μm, with a maximum error of 4.1%, and the established regression model has high precision and can accurately predict the machining results of laser-assisted turning of SiC ceramics. The optimization results of the orthogonal method and RSM show that the optimized process parameters obtained by the RSM are used for the laser-assisted turning experiment, and the measured surface roughness *Ra* value is 0.294 μm, which is 6.67% lower than that of the orthogonal method. So the RSM can obtain the smallest surface roughness, and more feasibly.
(4) The surface morphology analysis shows that compared with the traditional turning process, the machining effect of the laser-assisted turning process is better. Compared with orthogonal optimization, the surface roughness obtained by optimizing the process parameters of laser-assisted turning based on RSM is the smallest, there are no cracks and obvious defects on the surface, and the surface quality is significantly improved.

## Figures and Tables

**Table 1:** Performance indicators of SiC.

Density (g/cm^3^)	Hardness (HV)	Specific Heat Capacity (J/kg·K)	Thermal Conductivity (W/m·K)	Coefficient Thermal Expansion (°C)	Bending Strength (MPa)
3.15	2100	1100	80	4.5 × 10^−6^	400

**Table 2:** Orthogonal experimental factor level.

Parameters	Unit	Levels of Factors		
		Level 1	Level 2	Level 3
Laser power (P)	W	210	225	240
Cutting depth (a_p_)	mm	0.10	0.15	0.20
Rotational speed (n)	r/min	1500	1620	1740
Feed speed (f)	mm/min	2	3	4

**Table 3:** Response surface experimental factor levels.

Parameters	Notation	Unit	Levels of Factors		
			−1	0	+1
Laser power (P)	*A*	W	210	225	240
Cutting depth (a_p_)	*B*	mm	0.10	0.15	0.20
Rotational speed (n)	*C*	r/min	1500	1620	1740
Feed speed (f)	*D*	mm/min	2	3	4

**Table 4:** Orthogonal experimental results.

Value	Laser Power P/(W)	Cutting Depth a_p_/(mm)	Rotational Speed n/(r/min)	Feed Speed f/(mm/min)	Surface Roughness *Ra*/(μm)
1	1	1	1	1	0.417
2	1	2	2	2	0.546
3	1	3	3	3	0.765
4	2	2	1	3	0.374
5	2	3	2	1	0.682
6	2	1	3	2	0.337
7	3	3	1	2	0.538
8	3	1	2	3	0.321
9	3	2	3	1	0.375

**Table 5:** Analysis of variance of surface roughness.

Source	Degree-of-Freedom (DF)	Sum-of-Squares (SS)	Mean-of-Squares (MS)	F-Value
Laser Power (P)	2	0.042394	0.021197	84.30
Cutting depth (a_p_)	2	0.150289	0.075144	298.85
Rotational speed (n)	2	0.008388	0.004194	16.68
Error	2	0.201573		
Total	8			

**Table 6:** Range analysis of surface roughness.

Value	Laser Power P/(W)	Cutting Depth a_p_/(mm)	Rotational Speed n/(r/min)	Feed Speed f/(mm/min)
1	1	1	1	1
2	1	2	2	2
3	1	3	3	3
4	2	2	1	3
5	2	3	2	1
6	2	1	3	2
7	3	3	1	2
8	3	1	2	3
9	3	2	3	1
K_1_	0.5760	0.3583	0.4430	0.4913
K_2_	0.4643	0.4317	0.5163	0.4737
K_3_	0.4113	0.6617	0.4923	0.4867
*R*	0.1647	0.3033	0.0733	0.0177
Order	Cutting depth > Laser power > Rotational speed > Feed speed			

**Table 7:** BBD experimental results.

Value	Laser Power P/(W)	Cutting Depth a_p_/(mm)	Rotational Speed n/(r/min)	Feed Speed f/(mm/min)	Surface Roughness *Ra*/(μm)
1	0	0	−1	1	0.395
2	1	0	0	−1	0.361
3	0	0	0	0	0.405
4	0	1	1	0	0.719
5	−1	−1	0	0	0.451
6	1	1	0	0	0.523
7	−1	0	0	−1	0.523
8	−1	0	−1	0	0.515
9	0	−1	0	1	0.352
10	0	0	0	0	0.410
11	0	1	−1	0	0.645
12	1	0	−1	0	0.365
13	0	−1	0	−1	0.360
14	0	0	0	0	0.395
15	0	0	−1	−1	0.384
16	0	−1	1	0	0.337
17	0	0	1	−1	0.449
18	1	−1	0	0	0.312
19	−1	0	1	0	0.538
20	−1	0	0	1	0.530
21	0	0	1	1	0.463
22	1	0	1	0	0.345
23	−1	1	0	0	0.751
24	0	1	0	−1	0.689
25	1	0	0	1	0.323
26	0	−1	−1	0	0.346
27	0	1	0	1	0.668

**Table 8:** Analysis of variance of the regression model.

Source	Sum of Squares	Df	Mean Square	F-Value	*p*-Value	
Model	0.4379	14	0.0313	49.85	<0.0001	Significant
*A*-Laser power	0.0970	1	0.0970	154.61	<0.0001	
*B*-Cutting depth	0.2812	1	0.2812	448.15	<0.0001	
*C*-Rotational speed	0.0034	1	0.0034	5.37	0.0390	
*D*-Feed speed	0.0001	1	0.0001	0.1627	0.6938	
*AB*	0.0020	1	0.0020	3.16	0.1010	
*AC*	0.0005	1	0.0005	0.7367	0.4076	
*AD*	0.0005	1	0.0005	0.8068	0.3867	
*BC*	0.0017	1	0.0017	2.74	0.1235	
*BD*	0.0000	1	0.0000	0.0673	0.7997	
*CD*	2.250 × 10^−6^	1	2.250 × 10^−6^	0.0036	0.9532	
*A* ^2^	0.0017	1	0.0017	2.69	0.1269	
*B* ^2^	0.0479	1	0.0479	76.37	<0.0001	
*C* ^2^	0.0009	1	0.0009	1.50	0.2439	
*D* ^2^	0.0009	1	0.0009	1.39	0.2611	
Residual	0.0075	12	0.0006			
Lack of fit	0.0074	10	0.0007	12.71	0.0751	Not significant
Pure error	0.0001	2	0.0001			
Cor total	0.4454	26				
*R*^2^ = 0.9831			*R*^2^_adj_ = 0.9634			

**Table 9:** Validation of experimental results.

Case 1	Case 2	Case 3	Mean Value	Predicted	Error
0.293 μm	0.291 μm	0.297 μm	0.294 μm	0.282 μm	4.1/%

## Data Availability

No applicable.
